# The Glutathione System: A Journey from Cyanobacteria to Higher Eukaryotes

**DOI:** 10.3390/antiox12061199

**Published:** 2023-05-31

**Authors:** Corinne Cassier-Chauvat, Fanny Marceau, Sandrine Farci, Soufian Ouchane, Franck Chauvat

**Affiliations:** Université Paris-Saclay, CEA, CNRS, Institute for Integrative Biology of the Cell (I2BC), F-91190 Gif-sur-Yvette, France; corinne.cassier-chauvat@cea.fr (C.C.-C.); fanny.marceau@cea.fr (F.M.); soufian.ouchane@i2bc.paris-saclay.fr (S.O.)

**Keywords:** cyanobacteria, human, plants, glutathione, glutaredoxins, glutathione-S-transferases, iron-sulfur cluster, methylglyoxal, ergothioneine, ophthalmate, norophthalmate

## Abstract

From bacteria to plants and humans, the glutathione system plays a pleiotropic role in cell defense against metabolic, oxidative and metal stresses. Glutathione (GSH), the γ-L-glutamyl-L-cysteinyl-glycine nucleophile tri-peptide, is the central player of this system that acts in redox homeostasis, detoxification and iron metabolism in most living organisms. GSH directly scavenges diverse reactive oxygen species (ROS), such as singlet oxygen, superoxide anion, hydrogen peroxide, hydroxyl radical, nitric oxide and carbon radicals. It also serves as a cofactor for various enzymes, such as glutaredoxins (Grxs), glutathione peroxidases (Gpxs), glutathione reductase (GR) and glutathione-S-transferases (GSTs), which play crucial roles in cell detoxication. This review summarizes what is known concerning the GSH-system (GSH, GSH-derived metabolites and GSH-dependent enzymes) in selected model organisms (*Escherichia coli*, *Saccharomyces cerevisiae*, *Arabidopsis thaliana* and human), emphasizing cyanobacteria for the following reasons. Cyanobacteria are environmentally crucial and biotechnologically important organisms that are regarded as having evolved photosynthesis and the GSH system to protect themselves against the ROS produced by their active photoautotrophic metabolism. Furthermore, cyanobacteria synthesize the GSH-derived metabolites, ergothioneine and phytochelatin, that play crucial roles in cell detoxication in humans and plants, respectively. Cyanobacteria also synthesize the thiol-less GSH homologs ophthalmate and norophthalmate that serve as biomarkers of various diseases in humans. Hence, cyanobacteria are well-suited to thoroughly analyze the role/specificity/redundancy of the players of the GSH-system using a genetic approach (deletion/overproduction) that is hardly feasible with other model organisms (*E. coli* and *S. cerevisiae* do not synthesize ergothioneine, while plants and humans acquire it from their soil and their diet, respectively).

## 1. Introduction

Most life forms are continuously challenged with toxic reactive oxygen species (ROS) present in our oxygenic atmosphere (ozone, O_3_), and/or generated by respiration and cell metabolism [1,2,3] and photosynthesis in cyanobacteria [4,5,6,7,8], algae and plants [9,10,11]. In addition, photosynthetic organisms are exposed to solar UV that also generate ROS [12,13].

ROS molecules encompass singlet oxygens (^1^O_2_), superoxide anions (O_2_^●−^), hydrogen peroxides (H_2_O_2_), and hydroxyl radicals (^●^OH) that cause damages to target molecules, namely: lipids, nucleic acids and proteins [2,10], thereby generating cell death in microorganisms and multiple disorders and diseases in humans [14,15,16] that reduce longevity [17].

**Figure 1:**
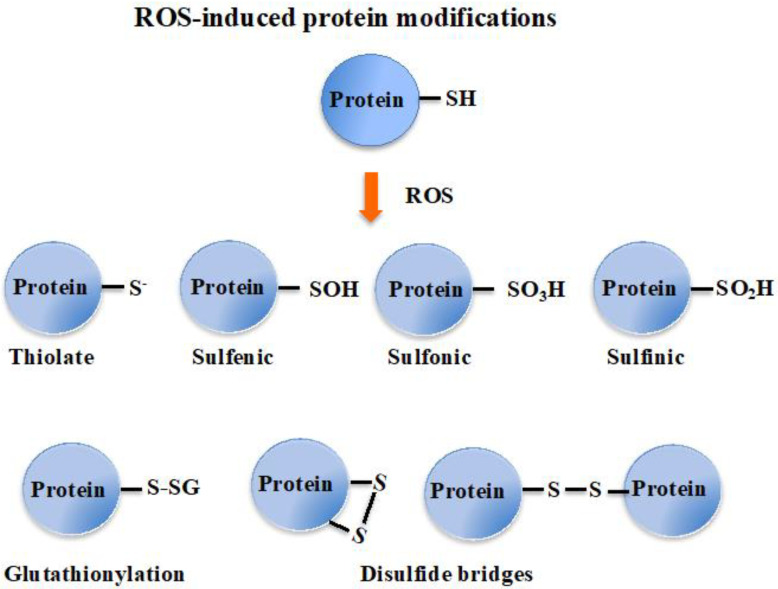
Schematic representation of the oxidation of the cysteinyl residue of protein to sulfenic (-SOH), sulfinic (-SO_2_H) and sulfonic (-SO_3_H); and disulfide (-S-S-) with another cysteinyl residue from the same or another protein, or a molecule of glutathione.

Superoxide anions and hydrogen peroxides can both react with proteins containing iron-sulfur [Fe-S] clusters, liberating their Fe ions. Free or complexed Fe^2+^ ions reduce H_2_O_2_, yielding hydroxyl radicals that modify all kinds of biomolecules at a diffusion-limited rate. Hence, radicals, sulfenic acids, disulfides and (hydro)peroxides are directly or indirectly formed by ROS [3,18]. ROS also oxidize cysteines to form thiyl (sulfenyl) radical (-S^●^) by one-electron transition; sulfenic acid (-SOH) and disulfide (-S-S-) by a two-electrons transition; sulfinic acid (-SO_2_H) by a four-electrons transition; and eventually sulfonic acid (-SO_3_H) by a six-electrons transition [19]. Concerning disulfides, two types can be distinguished considering whether they link two cysteinyl residues from either the same or different proteins (intra- or inter-molecular disulfide bridges), or from a protein and a molecule of glutathione (glutathionylation). Glutathione is the γ-L-glutamyl-L-cysteinyl-glycine tri-peptide (hereafter designated as GSH) that plays a prominent role in ROS detoxification from bacteria to higher eukaryotes [3,11,16,20,21]. It directly scavenges ROS and also serves as a redox cofactor for various antioxidant enzymes, such as glutaredoxins (Grxs), glutathione peroxidases (Gpxs), glutathione reductase (GR) and glutathione S-transferases (GSTs). The above-mentioned glutathionylation can protect cysteinyl residues against irreversible oxidation (generation of sulfinic and sulfonic acids), and/or act in regulation [9,11,18,22], as shown in Figure 1.

ROS can also be detoxified by various metabolites (ascorbate, carotenoids, vitamins, etc.) and several enzymes [23]. The superoxide dismutase (SOD) converts O_2_^●−^ to H_2_O_2_, which is then detoxified to H_2_O by the catalase and peroxidase enzymes [18]. H_2_O_2_ can also be detoxified by the hydroperoxide activity of some glutaredoxins [18]. The protein disulfides and glutathione-protein mixed disulfides are repaired by thioredoxins [20,24], glutaredoxins [3,11,25,26] and glutathione-S-transferases [14,16,25,27,28,29].

ROS-removing systems are usually viewed as beneficial antioxidants that maintain damaging ROS below dangerous levels [3,20,30]. However, ROS are also a necessary part of subcellular and intercellular communication in living organisms [3,18]. Indeed ROS species can serve as signal mediators in the redox regulation of cell metabolism [19], as they are enzymatically produced and degraded by NADPH-oxidases, which generate superoxide anions [3], SOD, which generates H_2_O_2_, and catalase and peroxidase, which detoxify H_2_O_2_ into H_2_O. Furthermore, H_2_O_2_ oxidizes protein thiols in disulfides or sulfenic acids, which can be reduced back to thiols, and are thereby good thiol redox switches for signaling [10,18]. Consequently, it has been proposed that “redox biology” [31] or “ROS processing systems” [10] would be a more accurate term than “(anti)oxidative systems” to describe cellular components that interact with ROS.

This review presents what is known concerning the evolutionary-conserved glutathione-system in selected model organisms, *E. coli*, *S. cerevisiae*, *A. thaliana* and human, emphasizing cyanobacteria for several reasons (See the next paragraphs for details and references). Cyanobacteria are environmentally crucial prokaryotes regarded as having evolved the oxygenic photosynthesis process, the chloroplast of algae and plants, and the glutathione-system to protect themselves against the ROS produced by their active photoautotrophic metabolism. Furthermore, cyanobacteria synthesize the thiol-less GSH homologs, ophthalmate and norophthalmate, and the ergothioneine antioxidant that operates in signaling and/or detoxication in humans. Moreover, cyanobacteria combine several important properties, such as (i) a simple nutritional requirement, (ii) a great physiological robustness, (iii) an important metabolic plasticity and (iv) the powerful genetics of some model strains. Hence, they are regarded as promising “low-cost” microbial factories for (i) the sustainable production of food and high-value chemicals for health and energy, (ii) the bioremediation of polluted waters and (iii) the fertilization of cultures.

## 2. Biological Importance and Biotechnological Interests of Cyanobacteria

Cyanobacteria are primordial prokaryotes regarded as the “inventor” of oxygenic photosynthesis [32], which played an important role in the evolution of Early Earth and the biosphere by absorbing a huge amount of the greenhouse gas carbon dioxide (CO_2_), and evolving a huge amount of dioxygen (O_2_) [33,34,35,36,37]. Indeed, cyanobacteria are regarded as responsible for the oxygenation (and oxidation) of the atmosphere since the Great Oxidation Event around 2.4 Ga [32,33,38,39,40,41,42].

As a consequence, cyanobacteria have long been challenged by ROS ^1^O_2_, O_2_^●−^, H_2_O_2_ and OH [2,3,18], which are generated by their active photosynthesis and, sometimes, their respiration [8,10,11]. Singlet oxygens are unavoidably produced by the interaction of sunlight with photosynthetic pigments (chlorophyll a, carotenoids and phycobiliproteins) while superoxide anions, hydrogen peroxides and hydroxyl radicals are generated when the light-driven electron transport exceeds what is needed for nutrients assimilation [8,10,43]. Cyanobacteria are also strongly exposed to solar UV radiations (UVR) that also generate ROS [20,44]. Consequently, cyanobacteria represent a major source of ROS in aquatic environments [45]. Furthermore, cyanobacteria were also the first organisms to cope with the oxygen-promoted changes in metal availability: decrease of iron (Fe), cobalt (Co), nickel (Ni) and manganese (Mn), and increase of zinc (Zn), molybdenum (Mo) and copper (Cu). This constitutes a real challenge because approximately one-quarter to one-third of all cellular proteins require metals [46].

Cyanobacteria are also frequently challenged by heavy metals (cadmium, cesium, chromate, mercury, lead, uranium, etc.) which are released by natural sources (volcanoes and forest fires) and anthropogenic activities (mining, fossil fuel burning, etc.). The toxicity of heavy metals is based on their chemical properties, which allow them to promote the production of ROS and the inactivation of enzymes [47,48,49], basically by reaction with SH groups, including that of GSH [50,51]. The presence of heavy metals in soils and waters is especially problematic because metals are persistent in the environment and they accumulate throughout the food chain, thereby threatening human health [46,52,53]. Cyanobacteria are important organisms to investigate the relations between metals and oxidative stress as they constitute the first biological barrier against entry of heavy metals into the food chain. Furthermore, cyanobacteria perform the two metal-requiring ROS-generating processes, photosynthesis and respiration, in the same membrane system [54,55]. Moreover, cyanobacteria are regarded as promising organisms for bioremediation of metal pollutants thanks to their robust photoautotrophic metabolism [55,56] and their multifarious mechanisms (biosorption, bioaccumulation and biotransformation) to sequester and minimize the toxic effects of heavy metals [57,58].

To cope with ROS and environmental stresses, cyanobacteria have evolved the glutathione system [5,59,60], which is crucial to their photoautotrophic lifestyle [4,44,61] and has been conserved during evolution [5,6,59,60,62]. The glutathione system comprises the glutathione tripeptide itself (γ-L-glutamyl-L-cysteinyl-glycine, GSH) and its cysteine-less homologs (ophthalmate and norophthalmate); in humans, these serve as biomarkers of diseases (See below), as well as numerous GSH-dependent enzymes, such as glutaredoxins and glutathione-S-transferases [18,63], which have been conserved during evolution [11,26,27,62,64,65]. In addition, cyanobacteria possess other promiscuous antioxidant enzymes, such as superoxide dismutases, catalases and peroxidases [8].

**Figure 2:**
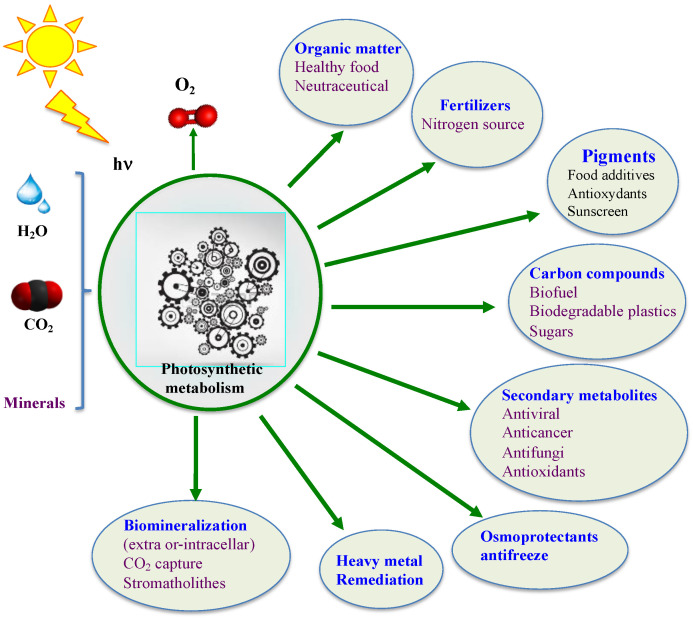
Schematic representation of the biotechnological interests of cyanobacteria. Minerals include calcium, nitrogen (N_2_, ammonium, nitrate and urea), phosphate, sulfate etc.

Contemporary cyanobacteria continue to play a key role in the global ecosystem. They fix enormous amounts of atmospheric CO_2_ and N_2_ to produce huge amounts of O_2_ and biomass for our food chain [32,33,36,37,56,66,67,68,69]. They have been consumed by humans and used as soil fertilizers for over a thousand years [70,71,72,73]. Furthermore, they produce a wealth of metabolites, such as vitamins, antioxidants (such as ergothioneine mentioned below), antibiotics, antifreezes, drugs, osmoprotectants and toxins [56,74,75,76,77] that can influence human health and/or improve plant growth and/or resistance to stress (drought, salt, heavy metals and pathogens) [78]. Currently, several cyanobacteria are being tested as a way to replenish O_2_, provide food, and recycle CO_2_ and urea wastes during long-term space missions [79,80]. Moreover, cyanobacteria are viewed as promising cell factories for the production of chemicals (biofuels, biodegradable bioplastics, drugs, solvents, etc.) from highly abundant natural resources: solar energy, water (fresh/marine), CO_2_ and minerals [74,75,81], thanks to their active and robust photoautotrophic metabolism [55,56] and the synthetic biology tools of model species [82], as shown in Figure 2.

Finally, in colonizing most aquatic ecosystems and soils of our planet, where they face various environmental challenges and interactions with competitors, predators or symbiotic hosts (angiosperms, bryophytes, fungi and gymnosperms), cyanobacteria have evolved as widely diverse organisms. They display various cell morphologies [83] and cellular differentiation [84], as well as widely diverse genome sizes (1.44–12.07 Mb), GC content (30–60%) and organization (presence of a circular chromosome with or without one to several linear chromosomes and circular plasmids) [82,85]. Hence, cyanobacteria are good model organisms to study the impact of environmental conditions and interactions with other organisms on the physiology, metabolism and morphology of microbial cells.

Together, the above-mentioned environmental importance of cyanobacteria and their interest for basic and applied science highlight the value of studying the glutathione system of cyanobacteria that has been conserved during evolution.

## 3. Synthesis and Importance of Glutathione in Living Organisms

**Figure 3:**
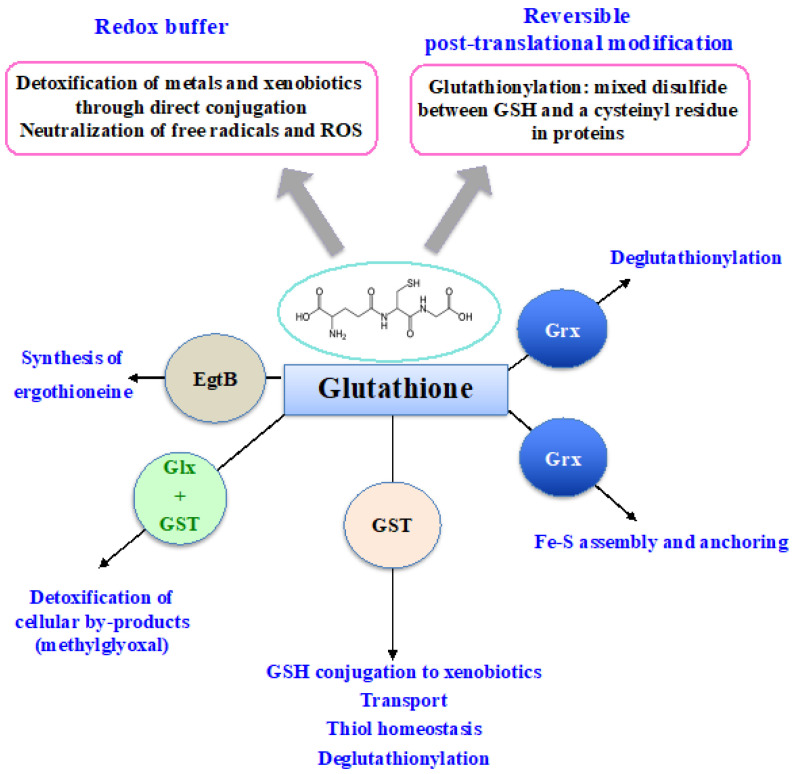
Schematic representation of the pleiotropic roles of glutathione.

Glutathione (GSH) was discovered in 1888 by J. de Rey-Pailhade, and its composition as γ-L-glutamyl-L-cysteinyl-glycine was established much later, in 1935 [86]. That the Cys and Glu of GSH are linked through the γ-carbonyl group of Glu instead of the typical α-carboxyl group confers a high stability to GSH since only very specific enzymes under particular conditions may operate on its degradation (See below). GSH is the most abundant non-protein thiol (concentration ranging from 0.1 mM to about 20 mM) in all three kingdoms of life: Bacteria (mostly Gram-negative, rarely Gram-positive), Archaea, and Eukarya, where it plays pleiotropic roles in cell life and resistance to stresses [3,5,11,15,18,20,21,87,88,89]. GSH is a nucleophilic metabolite that directly scavenges ROS, nitric oxides and carbon radicals [3,5,11,18]. GSH also serves as electron donor to various antioxidant enzymes, including glutaredoxins, glutathione peroxidases and glutathione-S-transferases (See below). Furthermore, GSH can also act in the synthesis of ergothioneine, another antioxidant catalyzed by the EgtB enzyme (See below). In addition, the cysteinyl thiols of GSH can complex metal [3,15,20], as shown in Figure 3.

GSH plays a prominent role in iron homeostasis in many prokaryotes [3,5] and most eukaryotes [18]. It is a key component of the cytoplasmic pool of labile iron, mostly occurring under the Fe(II)GSH complex [90], which likely supplies Fe for the synthesis of the Fe or [Fe-S] cluster cofactors of a wealth of enzymes involved in electron transfers (photosynthesis respiration) and central metabolism [15,18,88,91].

**Figure 4:**
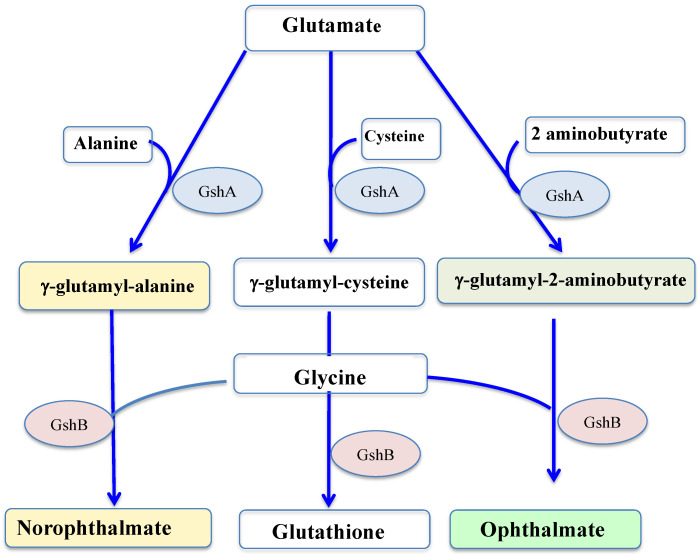
Schematic representation of the synthesis of glutathione and its thiol-less analogs. GshA: gamma-glutamylcysteine ligase; GshB: glutathione synthase.

GSH is synthesized by two sequential ATP-requiring enzymes, namely the γ-glutamyl-cysteine (γ-GC) synthetase (γ-GCS, EC 6.3.2.2), which forms γ-GC from L-glutamic acid and L-cysteine, and the GSH synthetase (GS, EC 6.3.2.3), which forms GSH from γ-GC and L-glycine, as shown in Figure 4.

In most cells, the two GSH-synthesis enzymes are encoded by separate genes, named *gshA* and *gshB* in prokaryotes and *gsh1* and *gsh2* in eukaryotes [9,20,92]. The evolutionary history of the GSH biosynthesis genes is complex in that the two genes in the pathway were acquired independently [60]. The gene encoding γ-glutamyl-cysteine ligase most probably arose in cyanobacteria [59] and was subsequently transferred to other bacteria and eukaryotes [5,6]. Then, eukaryotes and most bacteria apparently recruited a protein from the ATP-grasp superfamily of enzymes to synthesize glutathione from γ-glutamyl-cysteine and glycine [5,6,60]. In many organisms, the activity of γ-GCS (GshA), which is the rate-limiting step in the GSH biosynthesis pathway, is subjected to feedback inhibition by GSH to avoid over-accumulation of GSH [20,30,93].

Glutathione is vital in many organisms, including yeast [20,94,95], mice [96], plants [10,87,97] and cyanobacteria [4,61], but not in *Escherichia coli* [20,92,98,99]. *E. coli* mutants devoid of GSH do not exhibit enhanced sensitivity to oxidative stress (H_2_O_2_, cumene hydroperoxide, ionizing (gamma) radiations) in exponentially growing culture [92], but stationary-phase cultures are more sensitive to H_2_O_2_ than the wild-type [100]. However, an *E. coli* strain overproducing glutathione is more resistant to gamma-irradiations than the corresponding wild-type strain, not merely because it has a higher content of GSH per se, but because it has an increased capacity to synthesize GSH when irradiated [101]. It has also been shown that *E. coli* and *Salmonella typhimurium* accumulate reduced glutathione in the growth medium during the exponential phase [102] to protect cells against external toxic compounds such as H_2_O_2_, N-methyl-N′-nitro-N-nitrosoguanidine, iodoacetamide and heavy metals [103,104]. In microbial systems (bacteria and yeast) a specific glutathione uptake process exists to salvage glutathione from lysing cells [9].

In the yeast *S. cerevisiae gsh1*, mutants grow more slowly than the wild-type strain in rich medium and can grow in minimal medium only in the presence of exogenous GSH [20]. In contrast, *gsh2* mutants grow well in the absence of GSH and are not particularly sensitive to either H_2_O_2_ or *t*-butyl hydroperoxide. These results indicate that γ-glutamyl-cysteine, which accumulates in the *gsh2* strains, has some of the antioxidant activities of GSH [20].

In plants, where the complete absence of GSH causes death at the embryonic stage [97], mutants with a less severe decrease in GSH content are viable but more sensitive to many biotic and abiotic stresses [11], including Cd [105] and Zn [106]. The biosynthesis of γ-glutamyl-cysteine catalyzed by glutamate cysteine ligase takes place in chloroplasts [9], which likely originated from cyanobacteria [34,107,108], while the formation of GSH catalyzed by glutathione synthetase can occur both in the chloroplasts and in the cytosol. Then, GSH is transported to mitochondria and the nucleus [9,11,18,109]. In plants [109,110] and some cyanobacteria [111,112], GSH is also polymerized into phytochelatin to chelate metals that are coordinated by its numerous thiol groups. In addition, plants [113,114], cyanobacteria [115,116] and many other organisms [117] use the cysteine-rich protein metallothionein to chelate metals.

In humans, low levels of GSH and high levels of ROS are associated with HIV, diabetes mellitus and/or neurodegenerative diseases [16,118,119]. Cancer cells, with their high levels of GSH (and glutathione reductase activity, see below), are refractory to some of the therapies inducing oxidative stress. GSH can also react with the bioactive nitric oxide (NO) gas to produce S-nitrosoglutathione (GSNO), a storage form of this gaseous radical in tissues [89]. The reaction of NO with O_2_^●−^ to form peroxynitrite (ONOO^−^) conveys the GSNO-derived NO in the extracellular fluid. This process controls the physiological levels of this signaling molecule in tissues [120]. These properties likely explain the NO-like vasodilation properties of GSNO that are shared by other pharmacological NO donors [89]. In addition to GSH, human cells are protected from metal stress by synthesizing the metal-binding protein metallothionein [121], like many other organisms [117].

Some Gram-positive bacteria, including *Actinobacillus pleuropneumoniae* [122], *Listeria monocytogenes* [123], *Pasteurella multocida* [124] and both *Streptococcus agalactiae* and *Streptococcus thermophilus* [125,126], contain a newly discovered bifunctional enzyme, termed GshF, which possesses both GshA and GshB activities. The N-terminal sequence of GshF is similar to that of *E. coli* GshA, but the C-terminal sequence is more similar to the D-Ala, D-Ala ligase than to any known GshB [125]. Interestingly, GSH inhibits neither the GshA activity nor the GshB activity of the GshF, and the GshA activity of GshF is higher compared with that in other organisms, and it is not inhibited by GSH [127]. More than 20 bacteria, mostly Gram-positive, possess a *gshF*-like gene. Recently, the *S. thermophilus gshF* gene was overexpressed in tobacco plants, *E. coli* and yeast cells to increase their production of GSH, an objective of biotechnological interest [126,127,128,129,130].

Due to its critical role in antioxidation, xenobiotic detoxification, and immune regulation pathways, GSH has been widely used in the food, cosmetic, and pharmaceutical industries [131]. So far, GSH is commercially produced mainly by *Saccharomyces cerevisiae* strains, which have generally been recognized as safe. Previous studies have focused on overproducing GshA and GshB, but the production yield and titer of GSH in such *Saccharomyces cerevisiae* strains remain low due to the feedback inhibition on GshA [127]. To overcome this limitation, the GshF bifunctional enzyme from Gram-positive bacteria was produced in *S. cerevisiae*, as GshF is insensitive to feedback inhibition. The resulting strain produced 240 mg L^−1^ GSH with GSH content and yield of 4.3% and 25.6 mg_glutathione_/g_glucose_, respectively [127]. However, this production of GSH by *S. cerevisiae* competes with glucose demands of other industries and results in high production costs [131]. To save production cost, we think that this approach of using GshF for GSH production should be tested in cyanobacteria because they can produce high-value chemicals from sunlight and CO_2_ instead of glucose.

Instead of GSH itself, some organisms employ its precursors or derivates [3,59,120], such as γ-glutamyl-cysteine in halobacteria and halophilic archaea or trypanothione in kinetoplastid parasites [132]. They may also use other thiols, e.g., bacillithiol in Gram-positive Firmicutes [133] or mycothiol in many actinobacteria, such as the human pathogen *Mycobacterium tuberculosis* [134].

In some organisms, such as mycobacteria [135], the γ-glutamyl-cysteine peptide is also used for the synthesis of ergothioneine (hereafter EGT), an unusual thio-histidine betaine amino acid (also known as 2-mercaptohistidine trimethylbetaine) that has potent antioxidant and cytoprotective activities [136,137,138,139,140]. Hence, in mycobacteria there is competition between EGT and glutathione biosynthesis. EGT has both a thiol (antioxidant) and a thione form [136,140], with the latter thione tautomer being predominant at physiological pH, thereby making EGT unusually resistant to oxidation by molecular O_2_ [137]. Its midpoint potential, +0.06 V, is unusually high compared to typical thiols, including GST (−0.2 to −0.4 V) [137]. EGT can serve as a reductant via one-electron reaction or as a nucleophilic reagent via two-electrons exchange. In vitro studies have shown that EGT can scavenge ROS, such as singlet oxygen, hydroxyl radical and, more slowly, hydrogen peroxide H_2_O_2_. When EGT acts as a direct antioxidant (reductant), it is oxidized in EGT disulfide, EGT sulfenic or sulfinic acid, depending on the conditions (pH and/or the presence of thiols, the strength of oxidants, etc.) [136]. EGT-sulfinic is unstable and irreversibly degraded into L-hercynine, or oxidized to EGT sulfonic acid [139]. EGT can also participate in the chelation of divalent metals (Co, Cu, Hg, Ce, Pt) and in radiative reactions by physically deactivating high-energy molecules via energy transfer [137,139].

Few other organisms, such as cyanobacteria (see below) and certain fungi (*Neurospora crassa*, the fission yeast and mushroom fruiting bodies) are able to synthesize EGT [136,141,142], unlike plants and animals who acquire it via their soil and their diet, respectively [135,137]. EGT is stable in the body for a long time after ingestion, and is viewed as protecting the central nervous system against diseases [138]. Animals have evolved a highly selective transporter for it, originally known as a carnitine transporter (OCTN1) [138,140] and also called ergothioneine transporter ETT because of its 100-fold higher affinity for EGT [139]. Genetic analysis in mycobacteria [142] and EGT consumption in mammals has shown that EGT protects cells against oxidative [137,139], metal [143] and UV [137,139] stresses. Hence, EGT has been considered safe by regulatory agencies and may have value as a nutraceutical and antioxidant [137,139].

Returning to glutathione, some of its homologs have no cysteine residue, and therefore no reducing properties, such as ophthalmate (L-γ-glutamyl-L-α-aminobutyryl-L-glycine) and norophthalmate (L-γ-glutamyl-L-alanyl-L-glycine). Ophthalmate (hereafter OPH) and norophthalmate (NOPH) were initially discovered in various animal organs (lens, brain and liver) [144,145], where they are regarded as biomarkers of GSH depletion elicited by oxidative stress [146,147]. OPH was also found to have accumulated in stressed plants [148], yeasts [149], bacteria (*E. coli*) [150] and cyanobacteria (*Synechocystis* PCC 6803) [61]. Like GSH, both OPH and NOPH are synthesized by the GshA and GshB enzymes [61,146,147,148,149], as seen in Figure 4.

### Evolutionary Interest of Glutathione Synthesis in Cyanobacteria

Cyanobacteria contain a high concentration of intracellular GSH (2–10 mM), mainly in the reduced form [7,151,152], and they can accumulate GSH when supplied with GSH precursor amino acids (Glu, Cys or Gly), especially Cys [152], similar to what was observed in the yeast *Saccharomyces cerevisiae* [153]. Such cyanobacterial cells with a higher GSH content were more tolerant to heat [154]. Similarly, the unicellular model *Synechococcus elongatus* PCC 7942 that contains more GSH than the other unicellular model *Synechocystis* PCC 6803 is more tolerant to chromate than the latter cyanobacterium [155].

The activity of GshA from the model cyanobacteria *Anabaena* PCC 7120 [156] and *Synechocystis* PCC 6803 [6], as well as GshB from *Synechococcus elongatus* PCC 7942 [157], have been verified following their production in *E. coli*. GshA was found to be inhibited by both GSH and 1-buthionine sulfoximine [6,156], like most GshA enzymes [20,30,93]. GshA most probably arose in cyanobacteria and was subsequently transferred to other bacteria and eukaryotes, which then recruited GshB to synthesize GSH, like cyanobacteria [5,6,59,60]. Supporting this hypothesis, both *gshA* and *gshB* genes appeared to be essential in the model cyanobacterium *Synechocystis* PCC 6803 [4,61]. Furthermore, the GshB-depleted mutant was showed to be sensitive to O_2_ [61] and photo-oxidative stress [4,61], cadmium [158] and the antibiotic gentamicin [159].

Due to its importance for the food, cosmetic and pharmaceutical industries [131], GSH is commercially produced mainly by fermentation of *Saccharomyces cerevisiae* overproducing both GshA and GshB [127], or the more active GshF bifunctional enzyme [127]. However, this GSH production competes with glucose demands of other industries [131]. To save glucose costs, we propose to overproduce GshF in cyanobacteria, so as to produce GSH from sunlight and CO_2_. For this purpose, *Arthrospira* species (commercial name *Spirulina*) are of special interest because they are generally recognized as safe.

In agreement with the conservation of the GSH system from cyanobacteria to higher eukaryotes, cyanobacteria can polymerize GSH into phytochelatin ((γ-Glu-Cys)_2–11_-Gly) to chelate metals on its thiol groups [111,112,160], like plants [109]. Cyanobacteria also synthesize the cysteine-rich metal-chelating protein metallothionein [115,116], like many other organisms [117], including plants [113,114] and humans [121].

Cyanobacteria were also found to synthesize thiol-less γ-glutamyl peptides (γ-glutamyl-Ala, γ-Glu-2-aminobutyryl, γ- Glu-Leu, γ- Glu-iLeu, γ-Glu-Met, γ-Glu-Phe, γ-Glu-Thr) [61,161], in response to oxidative stress [61] or other slow-growth conditions [161], which are viewed as reservoirs of amino acids in cyanobacteria. Such γ-glutamyl dipeptides that differ from γ-Glu-Cys have been identified in eukaryotes, such as *Saccharomyces cerevisiae* [162] and mice [163], supporting the notion that the GSH system has been evolutionarily conserved from (cyano)bacteria to higher eukaryotes. In mammals, these γ-glutamyl peptides are regarded as being beneficial for human consumption [164], but also involved in inflammation, oxidative stress and/or glucose metabolism leading to cardio-metabolic diseases and/or diabetes [165,166].

Thiol-less analogues of glutathione, γ-Glu-Ala-Gly (NOPH) and γ-Glu-2-aminobutyryl-Gly (OPH), accumulated in cyanobacteria challenged by glucose-triggered metabolic and oxidative stress [61]. OPH was also found to be accumulated in stressed bacteria [150], yeasts [149] and plants [148]. The synthesis of OPH and NOPH in the cyanobacterium *Synechocystis* PCC 6803 was found to depend on GshA and GshB [61], as observed in mammals [18,147]. In mammals, OPH and NOPH are regarded as liver and heart markers of GSH depletion elicited by oxidative stress [146,147,148,149]. Glucose-stressed cyanobacterial cells accumulate not only OPH and NOPH, but also GSH [167]. Thus, OPH and NOPH are likely generated by the depletion of cysteine (not GSH per se) caused by its accelerated incorporation into GSH to cope with the increased GSH need for the detoxification of oxidants and glucose-catabolites (methylglyoxal, See below).

Many cyanobacteria synthesize large amounts of the EGT antioxidant [61,168,169,170,171], and fishes that feed, to some extent, on cyanobacteria are thus provided with plenty of EGT [137]. This finding is interesting because there is an increasing demand for EGT [135,140], and extracting it and chemically synthesizing it from edible fungi has a high cost and low yield [137,139]. The biosynthesis of EGT is well understood in *Mycobacterium* [135]. The SAM-dependent methyltransferase EgtD transforms histidine into histidine betaine. EgtA, the glutamate cysteine ligase, synthesizes γ-Glu-Cys. EgtB adds the thiol group of γ-Glu-Cys to the side chain of histidine betaine, which is transformed by EgtC into the histidine betaine cysteine sulfoxide metabolite. This intermediate is then converted to EGT by EgtE, the pyridoxal 5′-phosphate-dependent cysteine desulfurization enzyme [135,136]. All mycobacterial EGT-synthesis genes have been overexpressed in *E. coli*, leading to EGT production [135,172].

In EGT-producing cyanobacteria, which necessarily possess all EGT-synthesis genes, *egtB*, *egtC* and *egtD*, but not *egtE*, have been identified by sequence homology with their mycobacterial orthologs [171], and we have observed in the model species *Synechocystis* PCC 6803 that it is GSH itself, not its γ-Glu-Cys precursor, that serves for EGT synthesis [61]. We propose to use *Synechocystis* PCC 6803 to identify the possibly unknown EGT-synthesis genes as well as the molecular role and the selectivity/redundancy of EGT, GSH, OPH and NOPH, in signaling and/or detoxification of oxidative and metabolic stresses. Furthermore, we propose to increase the expression of all EGT-synthesis genes as an attempt to generate a cyanobacterial factory for cheap, high-level production of EGT from solar energy and CO_2_.

## 4. Glutathione Degradation

Because of its γ-linkage between the carboxyl group of glutamate and the amine group of cysteine, GSH cannot be degraded by genuine protease. Dedicated peptidases, γ-glutamyl transpeptidase (GGTs; E.C. 2.3.2.2) and/or γ-glutamyl cyclotransferases (γ-GCT or GGCTs, EC 4.3.2.9), catabolize GSH in bacteria and/or eukaryotes [9,11,21,110].

The GGT enzyme is conserved throughout all three domains of life [11,16,173]. Bacterial GGTs are generally soluble and localized in the periplasmic space or secreted in the extracellular environment [173], whereas eukaryotic GGTs are embedded in plasma or vacuole membranes [9]. GGT enzymes release the cysteinyl-glycine dipeptide and 5-oxoproline, a cyclized form of glutamate (Zhang and Forman, 2012). Then, the cysteinyl-glycine dipeptide is broken down into cysteine and glycine by specific Cys-Gly peptidases (including the leucine aminopeptidase (EC 3.4.13.18)) while the 5-oxoproline is converted into glutamate by the ATP-dependent 5-oxoprolinase (EC 3.5.2.9). The released glutamate, cysteine and glycine can be ploughed back into the synthesis of reduced GSH [9,11]. In *E. coli* and a few other bacteria, the tripeptidase (PepT) also acts in GSH degradation, and both the GGT- and the PepT-encoding genes are dispensable for cell growth under favorable laboratory conditions [9,21].

In plants, GSH degradation seems to be as important as GSH synthesis for sulfur metabolism [110]. The apoplastic and vacuolar GSH pools are degraded by GGTs, which cleave the γ-glutamyl moiety of GSH, GSSG and GS-conjugates, or transfer the Glu residue is transferred to amino acids to produce γ-Glu amino acids [9,11,110], which are beneficial for human consumption [164]. *Arabidopsis* possesses four GGTs. GGT1 and GGT2 are localized in the apoplast and degrade extracellular GSSG into Glu and Cys–Gly [110], similar to mammalian GGTs. The Cys–Gly dipeptide is further broken down by a dipeptidase into Cys and Gly. They are then translocated to the cytosol where they serve in the synthesis of protein and GSH, followed by a novel round of export/degradation in the apoplast [9]. *GGT3* is considered a pseudogene since its transcript encodes a protein lacking a sequence important for GGT catalytic activity. The *GGT4*-encoded isoform is present in the vacuole where it supports detoxification processes by degrading GSH conjugates formed by glutathione S-transferase following exposure to toxic xenobiotics [11,28,110]. GSH degradation mainly occurs in the cytosol [11]. In *Arabidopsis*, it involves three γ-glutamyl cyclotransferases (GGCTs, EC 4.3.2.9). They specifically hydrolyze GSH into a Cys-Gly dipeptide and 5-oxoproline, which are further broken down into Glu, Cys and Gly by (i) the Leu aminopeptidase 1 (EC 3.4.13.18) encoded by AtLAP1 and (ii) by the 5-oxoprolinase (5-OPase, EC 3.5.2.9) [11,110]. The pathogenic bacterium *Ralstonia solanacearum* injects many proteinic virulence factors in plant host cells, including an active GGCT enzyme that degrades intracellular glutathione, as a strategy to subvert plant defenses systems [11].

In mammals, where it was first reported, the GGT enzyme is a cell-surface protein that contributes to the extracellular catabolism of GSH, but it has no role in either GSH or γ-glutamyl-Cys transport back into cells [9]. Interestingly, GGT is used as a diagnostic marker for many human diseases [16,174]. Mammals also have both ChaC1- and ChaC2-type GGCT enzymes, which are expressed only under endoplasmic reticulum stress or constitutively, respectively. ChaC1 and ChaC2 have high specificity and comparable *K*_m_ values for GSH, but ChaC1 has 10- to 20-fold higher catalytic activity than ChaC2 [110].

### Glutathione Degradation in Cyanobacteria: An Overlooked Aspect of Their Metabolism

Little is known in cyanobacteria concerning the degradation of glutathione. Freshwater cyanobacteria possess one to four presumptive GGT-encoding genes that have not been studied yet, whereas marine species have no such genes. Furthermore, cyanobacteria have no *pepT* orthologous genes. In the cyanobacterium *Synechococcus elongatus* PCC 7942, the inactivation of a gene encoding a leucyl aminopeptidase (LAP) with cysteinyl-glycinase activity was found to decrease the tolerance to UV [44].

## 5. Glutathione Reductase

The function of GSH depends on the reactivity of its cysteinyl thiol group, which can complex metals, be alkylated to thioethers or oxidized to disulfides, thereby forming a glutathione disulfide dimer (GSSG) [3,15,20]. Four processes can remove GSSG after oxidative challenge: (i) ATP-driven export of GSSG [20]; (ii) degradation of GSSG by peptidase [9,11]; (iii) reduction of GSSG by the glutathione reductase (GR) enzyme (see below and Figure 2) or the thioredoxin reductase/thioredoxin (TrxR/Trx) couple [3,15,20] and (iv) the glutathionylation/deglutathionylation of proteins yielding RSSG and GSH [11,18,22]. Under normal conditions, GSH is about a 100-fold more abundant than GSSG [18,20,21]. For example, the GSH/GSSG molecular ratio is about 200 in *E. coli* cells growing in the rich standard-medium LB.

**Figure 5:**
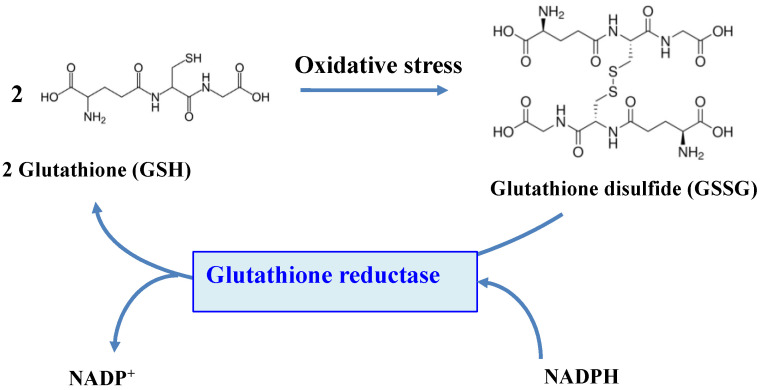
Schematic representation of the role of the glutathione reductase enzyme.

The flavoenzyme GR (EC 1.8.1.7) is a highly conserved enzyme across the tree of life, which converts GSSG to two molecules of GSH, by using NADPH (mostly) or NADH (rarely) as the reducing agents [11,15,18,20,21,175,176,177,178], as shown in Figure 5.

In vitro analyses showed that GR from humans, yeast and *E. coli* are inhibited by reduced glutathione [179].

GR belongs to the pyridine nucleotide-disulfide oxidoreductase family that includes the related enzymes dihydrolipoamide dehydrogenase, mercuric ion reductase trypanothione reductase and some TrxR-isoforms [3]. GR from pro- and eukaryotes share about 50% identity and form stable homodimers of ~110 kDa each comprising three domains [3,120,175]. The FAD- and NADP-(binding) domains are globular, whereas the interface dimerization domain is somewhat flat. It contains two regions, at the N-terminus 71–104 and C-terminus 372–482. The FAD-binding and the NADPH-binding domains are in residues 1–157 and 198–238, respectively. The catalytic site of GRs possesses two conserved cysteines (C61, C65) that can form a disulfide bond. In the *S. cerevisiae* enzyme, the cysteine C239 (not conserved) can bind excess GSH when required.

GR accumulate in cellular regions of high electron flux, where ROS are generated [120]. In prokaryotes, GR is localized in the periplasm, associated with the inner membrane facing the cytoplasm, and it can be secreted to the extracellular environment. In eukaryotes, GR is present in the cytoplasm, the endoplasmic reticulum lumen, the lysosomes and the organelles nucleus, mitochondria and chloroplasts, thanks to its transport from the cytosol [3,11,18,20,120,175,178].

In *E. coli*, GR deficient mutants, originally isolated in a screen for diamide sensitive mutants [180,181], have wild-type (WT) growth rates under standard conditions [20]. They are more sensitive than the WT strain to cumene hydroperoxide and the O_2_^●−^-generating compound paraquat, but not to *t*-butyl hydroperoxide [182,183]. Furthermore, their increased sensitivity to H_2_O_2_ could be uncovered only in a catalase mutant background [184]. Despite their weak GR activity (0.45 units as compared to 35 units in WT cells), the ratio of GSH to GSSG is not altered significantly from that of WT. This finding indicates that GSSG can also be reduced by other enzymes, such as thioredoxin reductase/thioredoxin (TrxR/Trx) couples [3,20,181]. In addition, an increased GSH synthesis in GR mutants may also help to maintain their high GSH levels [182].

In *Saccharomyces cerevisiae* and human cells, the GR-encoding gene was cloned on the basis of its sequence homology with the *E. coli* gene [20]. This eukaryotic GR gene expresses both the cytosolic and mitochondrial forms of GR, which are synthesized using alternative in-frame start codons. Starting at the first AUG codon, the synthesis generates a long GR isoform marked for transport to the mitochondria. The translation starting at the second AUG codon generates a shorter GR isoform remaining in the cytosol. Usually, the pre-sequence of the mitochondrial form is cleaved off upon import by mitochondrial proteases so that the mitochondrial and cytosolic forms have a similar length [20,120]. Yeast mutants lacking GR show WT growth, but accumulate increased levels of GSSG [185,186] and increased export of GSSG into the vacuole to maintain the highly reducing environment of the cytosol [18]. These mutants are very sensitive to H_2_O_2_ and the thiol oxidant diamide and are partially sensitive to cumene hydroperoxide, t-butyl hydroperoxide and paraquat [20,187]. Interestingly, GR-less mutants, which also lack the genes for thioredoxin 1 and thioredoxin 2, are nonviable under aerobic conditions and grow poorly anaerobically [186]. Thus, yeast cells require the presence of either the GSH- or the thioredoxin-dependent reducing systems for growth [185], as observed in *E. coli* (See above).

In humans, GR activity is positively correlated to longevity, and centenarians have an increased level of GR [17], but cancer cells in having high levels of GSH and GR are refractory to some therapies that induce oxidative stress [120]. Low GR activity is correlated with a higher susceptibility of cataract development during early adulthood [3] and HIV-1 infection [118]. In mice, GR was shown to act in the defense against bacterial infections [188].

In plants, GR activity is increased in response to abiotic stresses triggered by heavy metals, salts, drought, UV radiation and chilling temperatures [10,11]. Transgenic approaches elevating GR activity increased resistance to oxidative stress, whereas mutants with lower GR activities were more sensitive to oxidative stress and were affected in their development [3,10]. In *Arabidopsis*, GRs are encoded by two nuclear genes, *GR1* (*At3g24170*) and *GR2* (*At3g54660*), which are more expressed in roots and in photosynthetic tissues, respectively [11,120]. GR1 is present in the cytosol, nucleus and peroxisomes, whereas GR2 is dual-targeted to mitochondria [189] and chloroplasts [190]). Similarly, the two GR enzymes of the alga *Chlamydomonas reinhardtii* were shown to act in the protection against photo-oxidative stress [178].

Also interestingly, in vitro analysis of the two GR enzymes of the marine diatom *Thalassiosira oceanica* produced as recombinant proteins from *E. coli* were found to couple the oxidation of NADPH to the reduction of not only GSSG, but also oxygen, thereby generating superoxides in the absence of GSSG. As *Thalassiosira oceanica* is abundant in oceans, its GR activity is likely to be a contributor to the production of the large quantity of superoxides observed in oceans. As these ToGR are similar to GRs from bacteria, yeast and humans, it has been proposed that the counterintuitive production of ROS by GR could be widespread [191].

### Glutathione Reductase in Cyanobacteria

The GR enzyme of the filamentous cyanobacterium *Anabaena* PCC 7119 was analyzed in cell extracts [192] and purified as a dimeric flavin adenine dinucleotide-containing protein [193]. Its kinetic parameters are comparable to those of chloroplast enzymes, but its molecular weight is lower, similar to that of non-photosynthetic microorganisms [193]. However, it has three differences with respect to GRs from heterotrophic organisms: (i) a strong acidic character of the protein, (ii) an absolute specificity for NADPH and (iii) an optimum pH of 9.0 [193]. Furthermore, the activity of the *Anabaena* PCC 7119 GR is inhibited by sulfhydryl reagents, Zn^2+^ ions and heavy-metal ions, with GSSG behaving as a protective agent [193].

The GR enzyme of the other filamentous cyanobacterium *Anabaena* PCC 7120 has been purified directly or after production in a GR deficient *E. coli* strain, before or after addition of a hexa-histidine tag at the C-terminal of the protein to facilitate its purification [194,195]. Its amino acid sequence has 41 to 48% identity with GRs of *Escherichia coli, Pseudomonas aeruginosa*, pea, *Arabidopsis thaliana* and humans. Like most GRs, the *Anabaena* PCC 7120 GR uses NADPH as a cofactor, but its *K_m_* values for NADPH and GSSG are higher than those of other GRs [194,195]. The *Anabaena* PCC 7120 GR also shows significant activity when NADH is used as a reductant, in agreement with the finding that it carries the G*X*G*XX*G “fingerprint” motif (amino acids 173–178) [196] typical of NADH-dependent enzymes [176,177] instead of the GXGXXA consensus motif of NADPH-dependent GR [176,177]. Furthermore, the *Anabaena* PCC 7120 GR harbors (i) a Lys residue (Lys203) in place of an Arg residue involved in NADPH binding by other GRs, and (ii) an insertion of 10 amino-acid residues that form an extra loop near the entrance of the pyridine-nucleotide-binding site [196]. Removal of this loop increased the catalytic efficiency of the *Anabaena* PCC 7120 GR with NADPH by reducing *K*_M_, and with NADH by increasing *k*_cat_ [196].

Another GR has been purified from the other filamentous cyanobacterium *Spirulina maxima* [197]. Its amino acid composition was very similar to other GRs, and its optimum pH was 7.0. The *Spirulina maxima* GR is predominantly tetrameric, in equilibrium with a minor dimeric form. Its dissociation into dimers was observed at pH of 9.5 or in 6 mM urea. However, its equilibrium at neutral pH was altered by neither NADPH nor by disulfide reducing reagents [197]. Cyanobacterial GR activities were increased in response to unusual growth conditions (increasing the concentration of phosphate [197] or replacing nitrate by ammonium [194]) or stresses triggered by pesticides [198] and metals [199,200]. In addition to other players [201], GR was shown to protect the O_2_-sensitive nitrogen-fixing nitrogenase enzyme from oxidative stress in the cyanobacterium *Gloeocapsa* LB795 [202].

As compared to filamentous cyanobacteria, GR has been poorly studied in unicellular species, likely because two of the best-studied species, including for biotechnological purposes (photosynthetic production of high-value chemicals and bioremediation), *Synechocystis* PCC 6803 and *Synechococcus* PCC 7002, have no GR enzymes [82,203]. Interestingly, the insect *Drosophila melanogaster* has no GR enzyme, and it employs a thioredoxin reductase/thioredoxin system to reduce GSSG back to GSH [204]. It will be very interesting to study and compare the GSH system of both *Synechocystis* PCC 6803 and *Synechococcus* PCC 7002 with that of the other well-studied unicellular model *Synechococcus elongatus* PCC 7942 that contains a genuine GR [203]. As these three cyanobacteria are robust, it is conceivable that *Synechocystis* PCC 6803 and *Synechococcus* PCC 7002 could compensate the absence of GR by using a thioredoxin reductase/thioredoxin system yet to be identified, or by having an increased GSH synthesis and GSSG turnover as compared to *Synechococcus elongatus* PCC 7942.

## 6. Importance of the Evolutionary-Conserved Glutathione-S-Transferase Enzymes

As the reactivity of glutathione (GSH) with proteins, small molecules and xenobiotics can be low in vivo [18,205], GSH-dependent reactions are accelerated by enzymes, such as glutaredoxins (See below) and glutathione-S-transferases (GSTs).

Glutathione-S-transferases (EC 2.5.1.18) constitute a superfamily of enzymes that play prominent roles in specialized secondary metabolism and detoxication. The presence of GSTs in most living organisms highlights their ancient origin and the preservation of their functions during evolution [3]. GSTs are widely studied in eukaryotes because of their great relevance to human health [14,64,206,207,208,209], plant growth [28,210] and responses to stresses [27,63,211,212] and pathogens [213]. GSTs are less well studied in prokaryotes even though they act in bacterial protection against metabolite by-product (methylglyoxal, see below) [214] and pollutants such as polychlorinated biphenyls (PCBs), dichloroacetate and polycyclic aromatic hydrocarbons (PHA) [215,216,217].

**Figure 6:**
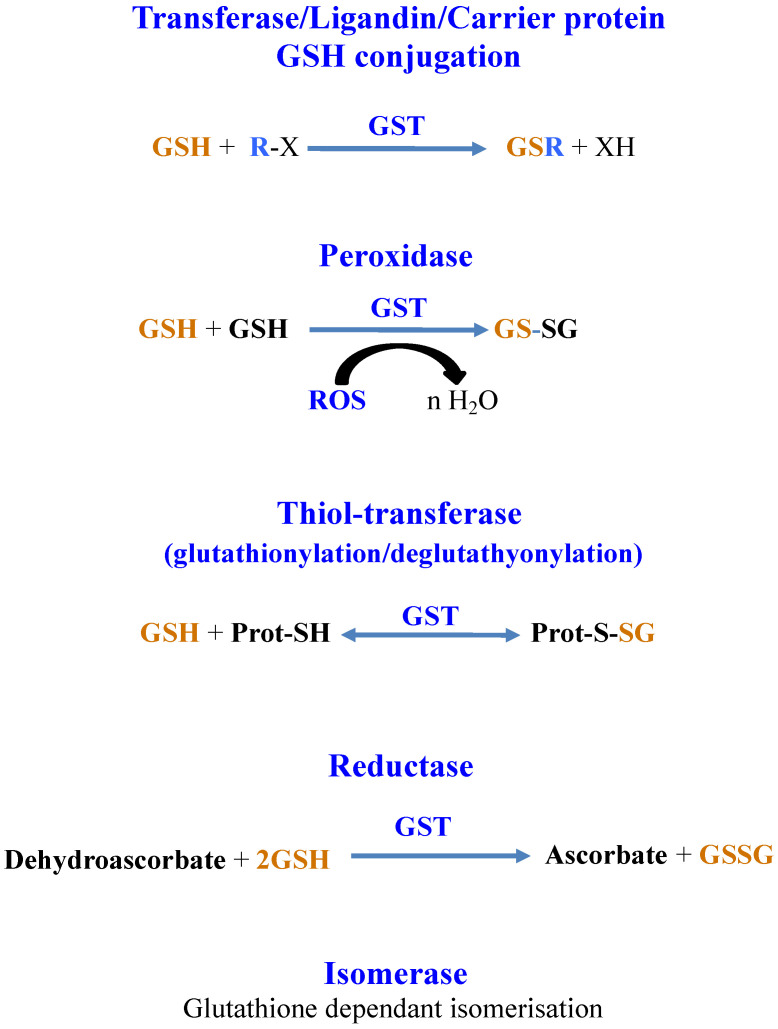
Schematic representation of the reactions catalyzed by glutathione-S-transferases.

GSTs catalyze reactions where GSH is consumed (GSH-conjugation on metabolites, chemicals or metals) or not (isomerization and dehalogenation) and reactions where GSH is oxidized (GSH-dependent peroxidases, -thiol-transferase, -dehydro-ascorbate reductase) [3,18]. GSTs can also bind and transport molecules through their noncatalytic ligandin properties [212,218]. Finally, GSTs can also interact with proteins to modulate their activity by glutathionylation/deglutathionylation (formation/reduction of a disulfide bridge between the cysteinyl residue of GSH and a cysteinyl residue of a target protein) [14,22,25,29], as shown in Figure 6.

GSTs are mainly homo- or heterodimeric enzymes, where each subunit contains an N-terminal thioredoxin (TRX) domain linked to an α-helical C-terminal domain [14,210,219]. The active site, located in a cleft between both domains, contains a GSH-binding site and a hydrophobic-substrate binding site. Based on their amino-acid sequence, GSTs were classified into various classes designated by a Greek letter [8,220]. GSTs having a sequence identity greater than 40% or lower than 25% belong to the same class or different classes, respectively [215]. GSTs are also grouped based on their localization in the cell, namely cytosolic, mitochondrial and microsomal, commonly referred as membrane-associated proteins in eicosanoid and glutathione metabolism (MAPEG) [3,18,62,215,217,219]. Finally, GSTs were further distinguished into four catalytic types, depending on an assumed important residue for catalysis, namely: tyrosine (TyrGSTs), serine (SerGSTs), cysteine (CysGSTs) and atypical (AtyGSTs) [8,14,210,217,221,222,223].

### Glutathione-S-Transferase in Cyanobacteria

The multiplicity of GSTs in plants (55 in *Arabidopsis thaliana* [28]) and humans (18 GSTs [206]) and their localization in one or several compartments (cytosol, chloroplast and/or mitochondria) [3,28,208] makes their analysis difficult. By contrast, cyanobacteria, the basic organisms that possess fewer GSTs, are attractive models for studying the selectivity/redundancy of these enzymes at the level of a whole organism. Furthermore, cyanobacteria are the primordial photosynthetic organisms and served as hosts for the evolution of GSTs with diversity in their structures, substrate recognition and catalytic functions [224].

We have recently started an analysis of all six evolutionarily conserved GSTs (designated as Sll0067, Sll1147, Sll1545, Sll1902, Slr0236 and Slr0605 [7]) of the unicellular model *Synechocystis* PCC 6803 that is well-studied for basic and applied research (bioremediation, bioproduction of high-value chemicals) [56,82]. Only Sll1545 appeared to be crucial to cell life [7,62,214], and our unpublished data. The rho class GST Sll1545 was found to have a good GSH-transferase activity with cumene hydroperoxide CuOOH [225], and to catalyze the detoxification of the water pollutant dichloroacetate [216], a toxic by-product of water chlorination and a metabolite of drugs [226]. We showed that Sll1545 and Slr0236 act defensively against photo-oxidative stress triggered by high light or H_2_O_2_ [7]. We also reported that the MAPEG-type GST, Sll1147 and its human orthologs, play a prominent role in the tolerance to membrane stresses triggered by heat, cold and lipid peroxidation [62]. That human orthologs of Sll1147 could rescue the stress tolerance of a *Synechocystis* PCC 6803 mutant lacking Sll1147 showed that the function of this MAPEG-type GST has been conserved during evolution from cyanobacteria to humans. The chi-class GST Sll0067 and its orthologs have been characterized biochemically from *Thermosynechococcus elongatus* BP-1, *Synechococcus elongatus* PCC 6301 and *Synechocystis* PCC 6803 [214,220,223,227]. Sll0067 purified from a recombinant *E. coli* strain as homo-dimer composed of about 20 kDa subunit appeared to have a good GSH-transferase activity with isothiocyanates (especially phenethyl isothiocyanate) and a low activity with the model substrate CDNB (1-chloro-2, 4-dinitrobenzene) [220,223]. Very interestingly, Sll0067 was found to play a crucial role in the detoxication of methylglyoxal ([214] and see below).

The halotolerant cyanobacterium *Halothece* PCC 7418 contains four putative GST [228]. One of them, H3557, is encoded by a salt-stress inducible gene and it exhibited the classical GST activity toward GSH and CDNB, which appeared to be salt tolerant [228]. Furthermore, recombinant *E. coli* cells producing H3557 exhibited an increased tolerance to H_2_O_2_ [228], in agreement with the amino-acids sequence similarity of H3557 with the rho-class GST Sll1545 of the model cyanobacterium *Synechocystis* PCC 6803 that protects it against H_2_O_2_ [7]. In *Anabaena cylindrica* and *Anabaena laxa* the GST activities were induced in response to the herbicide bentazone [229] and the fungicide R-metalaxyl, respectively [198]

## 7. Glutathione Acts in the Detoxification of Methylglyoxal, a By-Product of Cell Metabolism from Cyanobacteria to Humans

**Figure 7:**
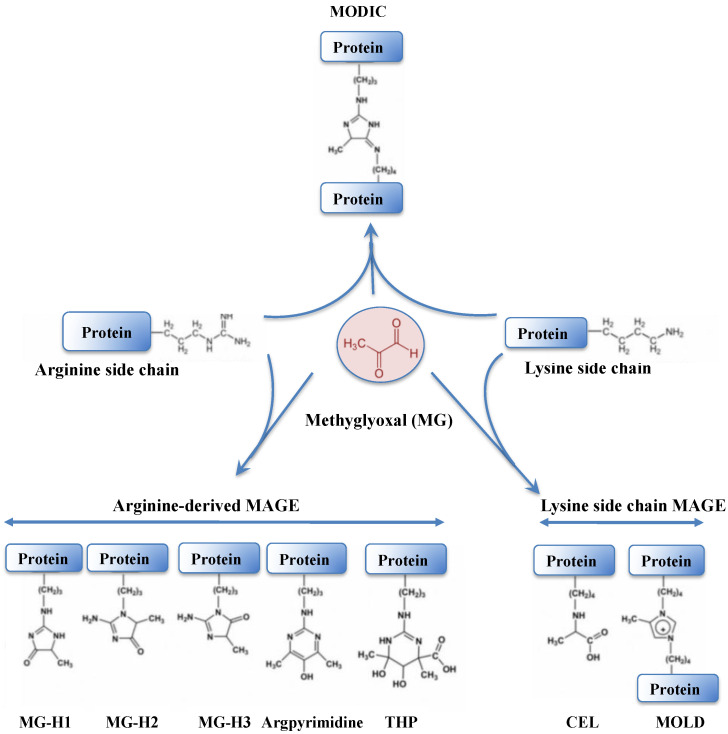
Schematic representation of protein modification resulting from the crosslinking of methylglyoxal onto arginine or lysine amino-acid residues. The abbreviations are as follows: MODIC, 2-ammonio-6-({2-[4-ammonio-5-oxido-5-oxopently)amino]-4-methyl-4,5-dihydro-1H-imidazol-5-ylidene}amino)hexanoate; MG-H1, N(delta)-(5-methyl-4-imidazolon-2-yl)-L-ornithine; MG-H2, 2-amino-5-(2-amino-5-hydro-5-methyl-4-imidazolon-1-yl)pentanoic acid; MG-H3, 2-amino-5-(2-amino-4-hydro-4-methyl-5-imidazolon-1-yl)pentanoic acid; THP, N(delta)-(4-carboxy-4,6-dimethyl-5,6-dihydroxy-1,4,5,6-tetrahydropyrimidine-2-yl)-L-ornithine; CEL, *N*^ε^-(carboxyethyl)lysine and MOLD: 1,3-di(*N*^ε^-lysino)-4-methyl-imidazolium.

Methylglyoxal (MG) is a very dangerous dicarbonyl compound that strongly interacts with lipids, nucleic acids and the arginine and lysine residues of proteins (Figure 7), generating advanced glycation end products (AGEs) that disturb cell metabolism in prokaryotes [230,231] and eukaryotes [230,232,233].

Like ROS, MG has a dual nature depending on its concentrations within the cells; it acts in signaling at low concentrations, but provokes detrimental effects, such as glutathionylation [234] (see below), at high concentrations [232,235]. In humans, MG is implicated in diabetes [236] and age-related disorders [230], such as retinopathy, nephropathy, cancer, and Parkinson’s and Alzheimer’s diseases [233,234,237]. Hence, MG is increasingly regarded as a marker of diabetes-related diseases. The calculated MG concentrations in mammalian cells were reported to vary between 0.5 and 5 μM, similar to what was observed in yeast (4 μM) [18]. In plants, elevated MG levels are a general response to abiotic and biotic stresses, such as salinity, heavy metals and drought [11]. Furthermore, MG is viewed as acting in signaling via abscisic acid, Ca^2+^, K^+^ and ROS, and these processes are regarded as useful for the development of stress-resilient crops [11,232,238].

MG is mainly formed in all cells both under normal and pathological conditions by the non-enzymatic breakdown of the triose phosphate isomers dihydroxyacetone phosphate (DHAP) and glyceraldehyde-3-phosphate (G3P) [230,231,233,234], which rapidly lose α-carbonyl protons and their phosphate groups, generating MG. MG is also generated by the spontaneous auto-oxidation of ketone bodies and sugars, lipid peroxidation and the Maillard reaction between reducing sugars and amino acids [239,240]. In addition, various enzymes generate MG from: (i) the fatty-acid derived acetone (cytochrome P450); (ii) the aminoacetone produced by the metabolism of glycine and threonine (monoamine oxidases); and (iii) the elimination of an inorganic phosphate from DHAP by the MG synthase in bacteria [236,241] and mammals [3,236].

MG is predominantly detoxified by the glyoxalase pathway, which starts by the supposedly “spontaneous” (non-enzymatic) conjugation of MG with GSH to form a hemithioacetal (HTA). HTA is then isomerized by glyoxalase I (GlxI, *S*-d-lactoylglutathione lyase; EC 4.4.1.5) to S-d-lactoylglutathione that is hydrolyzed by glyoxalase II (GlxII, *S*-2-hydroxyacylglutathione hydrolase; EC 3.1.2.6) to produce D-lactate and release GSH [3,11,18,236,237,242]. That GSH is required for MG detoxication explains why MG is accumulated in response to GSH depletion caused by oxidative stress [234]. GlxI belongs to vicinal oxygen chelate enzymes, which contain an ancient βαβββ-motif required for binding metal ions [3,234]. GlxI (also called Glo1) from humans, yeast and the parasite *Plasmodium falciparum*, is a dimeric protein that prefers Zn^2+^ (or Fe^2+^) at its active site, whereas the enzymes from *E. coli* and the pathogens *Yersinia pestis*, *Pseudomonas aeruginosa* and *Neisseria meningitidis* and the protist *Leishmania major* are optimally activated in the presence of Ni^2+^ (or Co^2+^) [237]. In *Arabidopsis*, overexpression of GlxI increased tolerance against salinity and maintained lower levels of MG as compared to the wild type (WT) plant [11]. GlxII (also called Glo2) is a monomeric protein composed of an N-terminal β-lactamase domain with a conserved Fe(II)Zn(II) center at the active site and a C-terminal domain with five α-helices [3,237]. In plants, GlxI and GlxII enzymes are encoded by multi-gene families, unlike what was observed in most prokaryotes and animals, which have single such genes [11]. In *E. coli* and yeast, both the *glxI* and *glxII* genes are non-essential to life, and growth phenotypes only became obvious upon challenge with exogenous MG [3,242].

MG can also be detoxified by other enzymes that do not require GSH, such as MG dehydrogenase, aldehyde dehydrogenases, aldo-keto reductases (AKR), α-dicarbonyl/L-xylulose reductase and the MG reductase [8,11,233,235,237]. AKRs, which exist across all phyla, are primarily NADP(H)-dependent monomeric oxidoreductases with a molecular weight ranging from 34 to 37 kDa [8].

### Methylglyoxal Detoxication: Lessons from a Cyanobacterium

Little attention has been paid to MG production, signaling and detoxification systems in photosynthetic organisms, although they produce MG by the catabolism of sugars, amino acids and lipids, such as heterotrophic organisms, but also by their active assimilation of CO_2_ driven by photosynthesis [54,232,243]. This issue is even more acute in cyanobacteria, the environmentally important prokaryotes [244], because they perform the two MG and/or ROS-producing pathways, photosynthesis (CO_2_ fixation and gluconeogenesis) and respiration (glucose catabolism), in the same cell compartment [54,55]. Furthermore, cyanobacteria are regarded as the inventor of oxygenic photosynthesis [33,34,35,37] and GSH and GSH-utilizing enzymes, such as glyoxalase (Glx) and glutathione transferases (GST), to cope with the ROS often produced by their active photosynthesis system [5,59,60].

In the model of cyanobacterium *Synechocystis* PCC 6803, we showed that (i) MG is toxic, (ii) both the *glxI* and *glxII* genes are non-essential to cell life but are required for the protection against MG and (iii) the MG synthase (EC.4.2.99.11.) gene is also non-crucial to cell growth [61,214]. We also reported that Sll0067 operates in the protection against MG, unlike the other five GSTs [7,62]. The Δ*sll0067* deletion mutant, which grew as fit the WT strain under standard (photoautotrophic) conditions, was found to be hypersensitive to MG. Furthermore, the MG-sensitive Δ*sll0067* mutant exposed to exogenous MG (or glucose) accumulated not only MG but also GSH, indicating that Sll0067 acts in an MG elimination process that requires GSH, similar to the GSH-dependent detoxification of MG catalyzed by the glyoxalase system. This interpretation was confirmed by in vitro tests showing that Sll0067 indeed catalyzes the conjugation of GSH with MG to form the hemithioacetal metabolite, which is known to be subsequently isomerized by GlxI and hydrolyzed by GlxII to release d-lactate and GSH [233,235]. Furthermore, the fixation of one MG molecule on the first subunit of the Sll0067 dimeric protein was found to stimulate the fixation of another MG molecule on the other Sll0067 monomer, thereby increasing Sll0067 activity [214]. The fixation of MG on Sll0067 also enhanced its affinity for GSH, and Sll0067 was also found to be activated by S-d-lactoylGSH, the intermediate metabolite in MG-detoxification. Collectively, these findings showed that MG enhances the Sll0067-driven conjugation of GSH and MG to promote MG detoxification by the glyoxalase pathway [214]. These data are important because, so far, the conjugation of GSH with MG was considered as spontaneous (non-enzymatic) in all organisms [232,233,235,237]. The finding that Sll0067 acts in the detoxification of MG, which causes diabetes in humans [231,233,234,237], is consistent with the correlation between the occurrence of diabetes and the poor activity of a human GST orthologous to Sll0067 [245]. These studies will certainly stimulate research on MG detoxification in mammals (possibly leading to the identification of biomarkers and/or drugs); plants (with interest for agriculture) and cyanobacteria (with interest for the sustainable production of valuable chemicals, such as lactate [246]).

## 8. Glutathione Maintains the Redox Homeostasis of Protein Thiols via Glutathionylation/Deglutathionylation Catalyzed by Glutathione-S-Transferases and Some Glutaredoxins

Oxidative stress promotes the covalent modification of proteins by GSH, i.e., formation of a disulfide bridge between the thiol group of a cysteinyl residue of a protein and a molecule of GSH [3,18,205]. This post-translational modification called S-glutathionylation is regarded as a transient protection of critical cysteines against irreversible oxidation towards sulfinic and sulfonic acid forms during oxidative stress [25,247]. It occurs only at specific cysteinyl residues of proteins, in response to ROS and MG [234,248], and not randomly. A basic environment or the proximity of a metal cation are key determinants for the tendency of thiol groups to become deprotonated and consequently be affected by oxidation and spontaneous S-glutathionylation [11]. As GSH is a bulky molecule, its ligation to proteins can have an impact on their structure, function, catalytic capacity and/or subcellular localization [11,22,25,29,207]. For example, three main glycolytic enzymes, GAPDH (glyceraldehyde-3-P-dehydrogenase) [249], aldolase and TPI (triose-P-isomerase) [250,251], are inhibited by S-glutathionylation under oxidative conditions in plants and other organisms, probably to redirect the glycolytic carbon flux towards the oxidative pentose phosphate pathway (OPPP) to generate reductive power in the form of NADPH [11].

GSTs [14,22,25,29,252] and glutaredoxins (Grxs) [22,25,26,248] catalyze both S-glutathionylation and deglutathionylation, while thioredoxins (Trxs) catalyze deglutathionylation [11,25,26].

Grx are small thiol proteins found in all kingdoms of life [3,10,18,22,26,29,253]. The first identified function of Grx was described as an electron donor for the ribonucleotide reductase enzyme (RNR) in a *E. coli* mutant lacking Trx [254], the classic hydrogen donor for RNR [255]. Grx can detoxify hydroperoxide thanks to their hydroperoxidase activity [18]. Bacterial Grxs have the most basic form of the Trx-fold, consisting of a four to five central β-sheet surrounded by three α-helices. Grxs of higher organisms frequently display additional N- and C-terminal helices. Interestingly, GSTs, the other glutathione-dependent enzymes, have similar architectures, supporting the theory of a common ancestor for Grxs and GSTs [3,256]. In the last two decades, the Grx family has impressively grown, and it has become clear that Grx is much more than a back-up system for Trx [257]. For example, in mammals at the physiological concentration of GSH, the GSH-Grx system sustains the RNR activity more efficiently than the Trxs [258].

Grx-isoforms can be structurally categorized as monomeric or dimeric proteins, which possess an active site with the sequence motif CXXC (dithiol Grxs) or CXXS (monothiol Grxs), with or without an Fe/S-cluster [3,18,26,88,91]. Grx can be furthermore grouped based on enzymatic activities, subcellular localizations or (putative) physiological functions (ROS detoxication, iron metabolism, etc.) [15,91]. In plants, Grx are involved in the regulation of development through interaction with distinct transcription regulators [10]. In humans, Grx functions have been implied in various physiological and pathological conditions, from immune defense to neurodegeneration and cancer development [26].

In general, CxxC-type Grxs function primarily in redox regulation and electron supply to metabolic enzymes. They catalyze the formation and reduction of disulfides, i.e., inter- and intra-molecular protein disulfides, including glutathionylation/deglutathionylation [15,22,25,26,29,234,252]. These redox-active Grxs often contain a consensus Cys-Pro-Tyr-Cys active site motif (CPYC-type of Grxs). The formation and reduction of protein disulfides require both their active site cysteinyl residues (dithiol reaction), while glutathionylation/deglutathionylation requires only their more N-terminal cysteinyl residue (monothiol mechanism). Both reactions start with a nucleophilic attack of the more N-terminal cysteinyl residue, which has a particularly low pK_a_ value, on the target disulfide. In the dithiol mechanism, the intermediate disulfide between the target protein and the Grx is reduced by its more C-terminal cysteinyl residue. The monothiol mechanism results in a reduced target protein, and a disulfide between the Grx and GSH. This disulfide can be reduced by another molecule of GSH, generating Grx and GSSG. Both reactions are fully reversible, as Grxs catalyze both the oxidation and reduction of their targets [15,18].

### Glutaredoxins and Glutathionylation in Cyanobacteria

Grxs are mostly studied in the model cyanobacterium *Synechocystis* PCC 6803 that possess only three Grxs: two CxxC-type Grxs, Grx1 and Grx2, and one CGFS-type Grx (Grx3), which are all dispensable to cell growth under standard conditions [203,259,260]. Both Grx1 and Grx2 act defensively against oxidative and metal stresses [49,203,260,261,262]. Interestingly, from the point of view of the selectivity/redundancy of these Grx, both Grx2 and Grx3, but not Grx1, were found to protect cells against H_2_O_2_, heat and high light [260]. Grx1, but neither Grx2 nor Grx3, was shown to physically interact with the mercuric/uranyl reductase enzyme MerA, which can be inhibited by glutathionylation, and subsequently reactivated by Grx1 [49]. Furthermore, Grx1, but not Grx2, was found to interact with NTR (NTR stands for NADPH-thioredoxin reductase). Interestingly, Grx1 and Grx2 were shown to act in an integrative redox pathway, NTR–Grx1–Grx2–Fed7 (Fed7 stands for ferredoxin 7) that protects *Synechocystis* PCC 6803 against selenate toxicity [203].

In *Synechocystis* PCC 6803, about 380 proteins involved in carbon and nitrogen metabolisms, photosynthesis, cell division and tolerance to stresses (GshB, Grx3 and GST sll1145) can be glutathionylated [263]. For four of these *Synechocystis* PCC 6803 proteins (the AbrB2 transcription regulator, the mercuric reductase, a peroxiredoxin and a 3-phosphoglycerate dehydrogenase), we have verified that their activity was indeed controlled by glutathionylation [49,263,264]. These data, together with similar findings obtained with other prokaryotes [26,247] and higher eukaryotes [11,14,22,25,26,249,250,251,265], showed that the glutathionylation/deglutathionylation regulatory process has been conserved during evolution.

## 9. Glutathione, Glutaredoxins and the Biogenesis of the Iron-Sulfur Cluster of Proteins

Glutathione plays a crucial role in cellular iron metabolism [15,90,266,267,268] and the synthesis and repair of iron-sulfur cluster [Fe-S] of a wealth of enzymes (See below). Iron (Fe) and sulfur (S) are crucial elements in all kingdoms of life [267,268,269]. Iron, the fourth most plentiful element in the Earth’s crust [270], is frequently a growth-limiting factor because ancient cyanobacteria raised the oxygen levels that oxidized the soluble ferrous ions (Fe^2+^) to insoluble ferric ions (Fe^3+^) [33,35,38,39,41,271,272]. Fe atoms are associated with many proteins as part of hemes, mono- or di-iron non-heme centers, or iron–sulfur [Fe-S] clusters [273,274]. Sulfur (S) is the fifteenth and the sixth most abundant chemical elements in Earth’s crust and aquatic environment, respectively [270]. Sulfur is essential in living organisms and is notably required for the synthesis of cysteine, which also serves for the synthesis of GSH, [Fe-S] and methionine [275]. Biological organisms absorb and assimilate sulfate from their environment via a reductive pathway involving a series of transporters, enzymes and GSH. This process is tightly controlled because it consumes energy and produces toxic compounds, notably sulfite and sulfide. In particular, it provides electrons to adenosine 5′-phosphosulfate reductases but also regulates the activity of glutamate-cysteine ligase by reducing a regulatory disulfide [275].

[Fe-S] clusters are critical cofactors in all categories of life. They participate in the transfer of electrons (photosynthesis and respiration), transcriptional and translation regulation, DNA repair and replication [15,18,26,88,91,266,273,276,277,278]. The chemically simplest Fe-S clusters are the rhombic [2Fe-2S] and the cubane [4Fe-4S] types, which contain iron (Fe^2+/3+^) and sulfide (S^2−^) [273]. [Fe-S] clusters were discovered in the early 1960s by purifying enzymes, including plant and bacterial ferredoxins, with characteristic electron paramagnetic resonance signals [273]. Later, chemists and biochemists devised in vitro protocols to assemble [Fe-S] clusters into apoproteins, and thereby assumed that these co-factors can assemble spontaneously on proteins in living cells. However, genetic, biochemical and cell-biological studies in the 1990s proved that the assembly and maturation of [Fe-S] centers on proteins is a catalyzed process rather than a spontaneous one [273].

[2Fe-2S] centers are synthesized from iron and cysteine-derived sulfur by highly conserved multi-protein machineries, including the iron-sulfur cluster (ISC) synthesis machinery [273,277,278] and many types of Grx [15]. The first [Fe-S]-Grx were isolated from humans (CSYC-type Grx, [279]) and the poplar tree (CGYC-type Grx, [280]). In both enzymes, the apo form (monomer) is a regular redox active Grx, while the holo form (dimer) has a bridging [2Fe-2S] cluster but no oxidoreductase activity. This [2Fe-2S] center lies at the interface of a dimeric complex of two Grxs ligated by the two N-terminal active site thiols and the thiols of two non-covalently bound GSH molecules [91]. In both CSYC- and CGYC-type Grxs, the exchange of the seryl or glycyl residue, respectively, for a prolyl residue abolished cluster ligation, in agreement with the absence of [Fe-S] cluster in natural CPYC-type Grx [15]. These CPYC-type Grx catalyze the GSH-dependent reduction of protein disulfides and deglutathionylation [91].

Following the discovery of these C(non-P)YC-type Grxs, CGFS-type Grxs have been characterized as [2Fe-2S]-proteins [15]. They occur as homodimer containing a subunit-bridging [2Fe-2S] cluster ligated by the catalytic cysteine of the CGFS motif of each monomer and the cysteines of two molecules of GSH from bacteria to higher eukaryotes [15,65,281]. With few exceptions, CGFS-type Grxs are inactive as oxidoreductases; they act in iron metabolism and [Fe-S] cluster formation and transfer [15]. Interestingly, the engineering of a CxxC-type Grx with a CGFS-type loop switched its function from oxidoreductase to [Fe-S] transferase, and the introduction of a CxxC-type loop into a CGFS-type Grx abolished its [Fe-S] transferase activity and activated the oxidative half-reaction of the oxidoreductase [91].

### Glutaredoxin and the Biogenesis of Iron-Sulfur Clusters: Lessons from Cyanobacteria

Cyanobacteria have a very high requirement for Fe (~10-fold more than non-photosynthetic prokaryotes) [271]. The electron flow associated with the operation of photosystems I and II (PS I and PS II) requires ~22 Fe atoms [270]. The unicellular model *Synechocystis* PCC 6803 contains the conserved gene clusters involved in Fe-S cluster biosynthesis: the *suf* (sulfur utilization factor) operon, some of the *isc* ([Fe-S] cluster) genes, and a single *nif* (nitrogen fixation) gene [278]. Only the gene products from the *nif* and *suf* operons are required for growth under standard photoautotrophic conditions [278]. The small NifU protein (SyNfu, 76 amino-acids) with a conserved CXXC motif harboring a [2Fe-2S] cluster serves as the principal scaffold protein required for iron-sulfur cluster biosynthesis and transfer to apo-ferredoxin [282]. SyNfu is also capable to deliver cluster to both the yeast monothiol Grx3 and the human dithiol Grx2, albeit at a lower rate [278]. Cluster exchange experiments showed that GSH can extract the cluster from holo-SyNfu, but the transfer is unidirectional [278].

We previously reported that the third Grx of *Synechocystis* PCC 6803, Grx3, exists as a monomeric apoprotein or a dimeric holoprotein. The dimer contains a subunit-bridging [2Fe-2S] cluster ligated by the cysteinyl residue of the CGFS motif of each Grx3 monomer and the cysteinyl residue of two GSH molecules [65,281]. Very interestingly, this feature has been conserved in Grx3 orthologs from bacteria [65,281,283], yeasts [65,284,285], plants [15,65,284,286,287,288,289] and humans [15,65,91,284,290], supporting the notion that Grx functions have been conserved throughout evolution.

## 10. Discussion

The glutathione system (GSH, GSH-derived metabolites and GSH-dependent enzymes), which plays pleiotropic roles in cell detoxication in most living organisms, is crucial to human food, health and longevity. However, the in vivo analysis of the multiple players of the GSH-system is difficult in plants and mammals because of their physiological and genetic complexities (slow development, aging, tissue specificity, cellular differentiation, sub-cellular compartmentation, traffic between organelles, multiple genes families, etc.). In contrast, the analysis of the selectivity/redundancy of each player of the GSH-system is easier in basic organisms, such as unicellular cyanobacteria, which are endowed with a small genome that is easy to manipulate. It is indeed important to thoroughly analyze the GSH system of cyanobacteria for the following reasons. Cyanobacteria are environmentally crucial and biotechnologically important organisms. They are regarded as having evolved the GSH system to protect themselves against the ROS produced by their active photosynthetic metabolism and solar UV. They synthesize the thiol-less GSH homologs ophthalmate and norophthalmate as well as the toxic metabolite by-product methylglyoxal (MG) that serve as biomarkers of several diseases in humans. In addition, cyanobacteria synthesize the GSH-derived metabolites ergothioneine (EGT) and phytochelatin, which play crucial roles in cell detoxication in humans and plants, respectively. Hence, cyanobacteria are well-suited organisms to thoroughly analyze the roles of the GSH system and its crosstalk with the ergothioneine player, using a genetic approach (deletion/overproduction) hardly feasible with other model organisms (*E. coli* and *S. cerevisiae* do not synthesize EGT, and plants and humans acquire EGT from their soil and their diet, respectively). Attesting the value of studying the GSH system of the model cyanobacterium *Synechocystis* PCC 6803, it was recently shown that (i) the CGFS-type glutaredoxins (Grxs) of *Synechocystis* PCC 6803, *E. coli*, *S. cerevisiae*, *A. thaliana* and humans harbor a GSH-ligated [2Fe-2S] cluster and (ii) the Sll1147 MAPEG-type glutathione-S-transferase of *Synechocystis* PCC 6803 and its human orthologs play a crucial role in the tolerance to oxidative stress. Furthermore, a *Synechocystis* PCC 6803 GST having orthologs in higher eukaryotes was shown to catalyze the conjugation of GSH on MG, the first step of MG detoxication that is always presented as spontaneous (not catalyzed by an enzyme) from bacteria to humans. These results show that cyanobacteria can indeed be used to characterize the evolutionarily conserved functions or features of actors of the GSH system. Therefore, we argue in favor of a comparative analysis of the GSH system of the three robust model cyanobacteria *Synechocystis* PCC 6803, *Synechococcus* PCC 7002 and *Synechococcus elongatus* PCC 7942 because they have interesting differences: *Synechococcus elongatus* PCC 7942 possesses a glutathione reductase (GR) enzyme but does not synthesize EGT, whereas the contrary is true for *Synechocystis* PCC 6803 and *Synechococcus* PCC 7002. As these three cyanobacteria are robust, it is conceivable that *Synechocystis* PCC 6803 and *Synechococcus* PCC 7002 compensate the absence of GR in using an as yet unknown thioredoxin reductase/thioredoxin system, similar to what was observed in *D. melanogaster*. Alternatively, *Synechocystis* PCC 6803 and *Synechococcus* PCC 7002 could compensate their lack of GR by having a very active GSH synthesis and/or GSSG turnover as compared to *Synechococcus elongatus* PCC 7942.

## 11. Conclusions

We have shown in this study that cyanobacteria, the basic organisms regarded as having invented the glutathione system for cell detoxication, are well-suited organisms to study the selectivity/redundancy of the evolutionarily conserved players of this system, which are important for agriculture and medicine.
